# How many children have palliative care needs and which of them are less likely to be actively seen by services? A cross-sectional analysis of survey data

**DOI:** 10.1136/bmjpo-2025-004069

**Published:** 2026-04-10

**Authors:** Maddy French, Catherine Walshe, Alex Garner

**Affiliations:** 1 International Observatory on End of Life Care, Division of Health Research, Lancaster University, Lancaster, UK; 2 Lancaster Medical School, Lancaster University, Lancaster, UK

**Keywords:** Palliative Care, Epidemiology, Child, Health services research, Noncommunicable Diseases

## Abstract

**Background:**

The prevalence of children with life-limiting conditions is increasing. It is difficult to estimate palliative care needs in this population, and few studies have explored the characteristics or service use patterns of this group. The aim of this study was to estimate the number of children with palliative care needs in North West England, exploring associations between patient characteristics and service activity.

**Methods:**

All services providing healthcare to children in North West England were surveyed to provide data on children (0–19 years) with palliative care needs within a census period (6–15 February 2023). Data collected included demographic information, diagnosis and care patterns. Point prevalence of need was calculated for all children by age group and ethnicity. Associations between patient characteristics (eg, age, ethnicity) and being actively seen by services were explored in logistic regression models.

**Results:**

49 services provided data on 1694 children. Point prevalence of need was 10.1 per 10 000 0–19-year-olds (95% CI 9.6 to 10.6). Children of South Asian ethnicity were less likely to be actively seen (defined as seen or called within the census period) than those of White ethnicity (OR 0.45, 95% CI 0.32 to 0.64).

**Conclusions:**

Disparities in the use of children and young people’s palliative care services were demonstrated. Future research should focus on developing measures of complex need for palliative care for children with life-limiting conditions, as well as inclusive and appropriate interventions to address disparities.

**WHAT IS ALREADY KNOWN ON THIS TOPIC**

- The prevalence of children with life-limiting conditions is increasing. Methods of estimating the need for palliative care among this group rarely account for the complexity of need or prognosis. Few studies have looked at the characteristics of children actively being seen by services.

**WHAT THIS STUDY ADDS**

- The population level need for children’s palliative care can be estimated using a prognosis-based framework of needs. Disparities in the use of children’s and young people’s palliative care services were found for some minoritised ethnic groups.

**HOW THIS STUDY MIGHT AFFECT RESEARCH, PRACTICE OR POLICY**

- This study highlights the need for careful and inclusive development of interventions that ensures all children and their parents, regardless of background, have access to safe, appropriate palliative care.

## Introduction

 The prevalence of children with life-limiting conditions has increased globally, and where projections are available, there are indications that it will continue to increase in the years to come.^1^,^7^ To enable effective and timely commissioning and provision of healthcare services for this population, it is necessary to estimate population needs and identify any potential gaps in provision. Existing methods of estimating population-level need have tended to use routinely collected data, such as diagnosis and hospital attendance.^2 8 9^ While providing a valuable overview, such data can be vulnerable to missingness, particularly data on characteristics such as ethnicity, or fail to take into account those who primarily receive care in other settings.^9^ Variability in the severity and prognosis of different life-limiting conditions among children also means it is difficult to estimate palliative care needs in this population.

A recent review has categorised approaches to defining and categorising the need in this population.^9^ Most studies are based on diagnostic categories or mortality data, with few taking into account symptomatology or any assessment of complexity.^9 10^ Those that do have such data were typically single-centre studies, only assessing the need in one type of setting.^9^ One different approach to identifying children with potential palliative and end-of-life care needs is to ask healthcare professionals caring for them a variation of the ‘surprise’ question (‘Would you be surprised if this child eventually died due to their underlying condition?’).^11^ The Spectrum of Children’s Palliative Care Needs is a prognosis-based framework based on this approach.^11^ To date, the Spectrum framework has not been used to try to estimate population-level need for palliative care. Using the framework for this purpose offers a complementary and potentially more sensitive method of estimating need than that determined through diagnostic codes.

Across the UK, there are 18 children’s palliative care networks that bring together service commissioners and other organisations involved in caring for babies, children and young people with palliative care needs. The North West Regional Children and Young People’s Palliative Care Network (‘the Network’) is a network representing the needs of children and young people across North West England. The Network includes representatives of babies, children and young people with palliative care needs and their families, commissioners and providers of children’s palliative care from the the National Health Service (NHS), voluntary sector, social services and education, across primary, secondary and tertiary care. The broad service coverage of this network provides an opportunity to explore the use of services across a large region of England.

## Methods

### Design

The aim of this study is to estimate the numbers and describe the characteristics of babies, children and young people (aged 0–19 years) with life-limiting illness who currently receive care within North West England using the Spectrum of Children’s Palliative Care Needs framework. The Spectrum of Children’s Palliative Care Needs is a classification framework for children with life-limiting or life-threatening conditions. It is designed for multidisciplinary teams to assess the likely prognosis of a child and group them accordingly, ranging from Green, where survival to adulthood is considered likely, to Red, where survival is not expected beyond the next few weeks. A survey design was used to capture information about babies, children and young people up to the age of 19 years with potential palliative and end-of-life care needs in the North West. All services known to the network were requested to complete the outline data on every child identified as having palliative or end-of-life care needs and receiving care (an active contact) within a given census period (6–15 February 2023).

### Objectives

The objectives of this study were threefold:

1. Describe the characteristics of children on the caseload of services who have palliative care needs using the Spectrum framework in North West England.
2. Estimate the point prevalence of palliative care need among this population.
3. Measure associations between patient characteristics and odds of being seen by services in the census week.

### Setting

The study area is the catchment area of the Network, which includes services in Greater Manchester, Lancashire, Cumbria, Merseyside and Cheshire (an approximate total population of 7 million people) in the North West of the UK. All services known to the Network (including specialist children’s hospitals, general hospitals with paediatric wards, community services, children’s hospices and specialist school provision) across this geography were invited to participate. This includes children’s community nursing teams, paediatric oncology outreach/Macmillan nursing teams, special school nursing teams, children’s hospices (inpatient and community), children’s continuing care teams, children’s inpatient and day case wards, including paediatric intensive care, neonatal intensive care units and special care baby units. General practices and accident and emergency departments were excluded.

### Population

Eligible babies, children and young people were those aged between 0 and 19 years (including those with a prenatal diagnosis) for whom the answer to the question ‘Are there any babies, children or young people currently on the ward/caseload for whom you wouldn’t be surprised if they eventually died due to their underlying condition?’ was ‘yes’.^11^ Hereafter, ‘child’ will be used when referring to the whole population of the study.

### Sampling and recruitment

All services known to the Network were invited to participate; there was no sampling of services. The approach to services inviting them to participate was coordinated by network staff who contacted key contacts within each service.

### Data collection

Data were collected on a pro forma basis that allowed details of each eligible child to be entered as a row on a spreadsheet. Each service identified a data collection lead who was responsible for coordinating the entry of data during the census week (6–15 February 2023). Where clinicians knew the child for whom they were inputting data and were confident in estimating their prognosis, they would complete this field themselves; otherwise, they would consult with another clinician in that team who could provide that information. All leads and those responsible for data entry received training to facilitate consistency in the data collection approach. The data collected are described in table 1. Data were anonymised before being shared with researchers, including transforming date of birth into age, NHS number into a study-specific code and postcode into Lower Layer Super Output Areas.

**Table 1:** Data collected

Data	Comments
National Health Service (NHS) number*	This was transformed into a pseudonymised code prior to secure transfer to Lancaster University. This allowed linkage where children appeared more than once in the dataset.
Date of birth*	This was transformed to an age in days, months and years prior to secure transfer to Lancaster University.
Sex	Male, female, other, unknown, NA.
Ethnicity	Black, East Asian, South Asian, Mixed, White, Other, Unknown, NA.
Postcode of residence*	This was transformed into an LSOA prior to transfer to Lancaster University and into information on ICB and local authority area. Postcode was used to obtain area deprivation ranks from the 2019 Index of Multiple Deprivation.^30^
Category of diagnosis	Neurology, haematology, oncology, metabolic, respiratory, circulatory, gastrointestinal, genitourinary, perinatal, congenital, other, unknown, NA.
Spectrum of needs colour	Red, Amber (years), Amber (months), Yellow, Green (for children ≥1 year). Red, Amber (months), Amber (weeks), Yellow, Green (for babies ≤1 year).
Has the child/family been seen or called during the census period?	Yes or no.
For community services: how often did the patient/family telephone?	More than once a day, daily, several times a week, weekly, monthly, less than once a month.
How often did the patient/family visit?	More than once a day, daily, several times a week, weekly, monthly, less than once a month.

* Data marked were anonymised prior to transfer to Lancaster University for analysis, according to agreed data management plans.

ICB, Integrated Care Board; LSOA, Lower Layer Super Output Areas.

### Missing or conflicting data

Clinicians completing the survey input data from medical records, supplementing this with their own knowledge of the child, many of whom had been under their long-term care. This resulted in generally low levels of missing data. However, all variables included a category of ‘Unknown’, which survey completers could use when information could not be gathered from medical records or from their own knowledge. The number and percentage of the study population with ‘Unknown’ data are reported in table 2 in the Results section.

Each child or baby in the dataset had a unique ID, allowing us to identify where multiple survey completers had provided data on individual patients. Where there were conflicting data provided by different completers, a single value was selected based on a set of criteria for each variable (see online supplemental table S1). The number and percentage of conflicting data reclassified as ‘Unknown’ in the final dataset is provided in online supplemental table S2.

### Data on babies

While the intention had been to look at babies (<1 year) in a separate analysis, the small numbers of babies seen prevented this. Babies were included in the overall analysis of point prevalence and associations between characteristics and whether patients were seen in the census week. The colour-coded level of need based on the Spectrum of Children’s Palliative Care Need is not reported for babies due to issues with data quality. While there was a separate Spectrum of Children’s Palliative Care Need framework for babies included in the survey, this was not used consistently, with some survey completers using the older children’s framework to estimate levels of need for babies.

### Analysis

The unique ID number ascribed to children meant that we were able to identify when children appeared multiple times in the dataset and link these data. Descriptive statistics were calculated, including aggregated numbers and proportions. Where cell numbers are ≤5, categories are collapsed or numbers are reported as ≤5 as per standard practice to avoid potential identification. All percentages are rounded to whole numbers.

Point prevalence of paediatric palliative care need across the North West of England was calculated using the study population as the numerator and the estimated number of children and young people aged 0–19 years using the Office for National Statistics’ mid-year population estimates for 2020 as the denominator.^12^ Prevalences were also broken down by age group. Estimates of prevalence in different ethnic groups in the North West were also calculated using population estimates of different ethnicities from an updated version of the ETHPOP (Ethnic Population Projections) database.^13^ Prevalences were mapped geographically across the North West at the Middle Layer Super Output Areas (MSOA) level, according to the place of residence of the child.

To understand factors associated with patients being seen by service providers, logistic regression models were fitted with whether a patient was seen or called during the study period as the response variable. Univariate models were first fitted, then a multiple logistic regression model was fitted containing all variables included in each univariate model. Explanatory variables that were tested were the spectrum of palliative care needs, colour, diagnosis category, ethnicity, index of multiple deprivation, age and sex. Model coefficients were used to calculate unadjusted and adjusted ORs of whether a patient is seen or called during the study period. We used a simple Bonferroni multiple testing correction to adjust our testing significance level α from 0.05 to 0.0006.

### Patient and public involvement

The study topic and research question were generated by practitioners working in the Network. While no patient and public involvement was undertaken before the study, findings were presented to representatives of the Network, including parents of children who had been under the care of children’s palliative care services in North West England.

## Results

Data were received from 49 services (four services did not participate, three in Cheshire and Merseyside and one in Lancashire and South Cumbria). Information was collected for about 1694 children in total. The number of children (>1 year) in the dataset was 1572, and babies (<1 year) were 121 (there was one child for whom age was not provided).

Characteristics of the children in this dataset are described in table 2. The age category with the highest percentage (27%) of children was 6–10 years. 54% of the children were male and 44% were female. The most common diagnosis category was neurological (33%), followed by respiratory (13%) and circulatory (10%).

**Table 2:** Characteristics of children and babies with palliative care needs in North West England and univariate associations between patient characteristics and odds of being seen in the census week

Characteristic	Total number (%)	Number seen in census week* (percentage of total number)	Univariate	
			OR (95% CI)	P value
Spectrum of Children’s Palliative Care Need (for those measured using the children’s version of the Spectrum framework)†				
Red	27 (2)	17 (63)	2.45 (1.12 to 5.64)	0.03
Amber (months)	121 (8)	88 (73)	4.22 (2.72 to 6.70)	<0.01
Amber (years)	381 (24)	228 (60)	2.23 (1.71 to 2.93)	<0.01
Yellow	473 (30)	188 (40)	1.00 (0.77 to 1.29)	0.94
Green	564 (36)	219 (39)	(Baseline)	
Age‡				
0–12 months	121 (7)	103 (85)	4.44 (2.66 to 7.81)	<0.01
1–5 years	442 (26)	246 (56)	(Baseline)	–
6–10 years	463 (27)	213 (46)	0.70 (0.54 to 0.91)	<0.01
11–15 years	396 (23)	167 (42)	0.64 (0.49 to 0.85)	<0.01
16–18 years	253 (15)	107 (42)	0.60 (0.43 to 0.82)	<0.01
19+ years	18 (1)	12 (66)	1.55 (0.59 to 4.53)	0.39
Sex				
Female	747 (44)	377 (50)	(Baseline)	
Male	910 (54)	447 (49)	0.96 (0.79 to 1.17)	0.69
Other/unknown	37 (2)	25 (65)	2.37 (1.12 to 5.48)	0.03
Ethnicity				
Black	54 (3)	31 (57)	1.75 (0.95 to 3.37)	0.08
East Asian	42 (2)	21 (50)	0.85 (0.46 to 1.58)	0.60
Mixed	36 (2)	18 (50)	0.95 (0.48 to 1.91)	0.89
Other	53 (3)	27 (51)	1.09 (0.61 to 1.97)	0.77
South Asian	268 (16)	88 (33)	0.48 (0.36 to 0.63)	<0.01
Unknown	98 (6)	53 (54)	1.02 (0.67 to 1.55)	0.92
White	1143 (67)	611 (53)	(Baseline)	
Diagnosis				
Neurology	562 (33)	284 (51)	(Baseline)	
Respiratory	224 (13)	97 (43)	0.69 (0.50 to 0.94)	0.02
Congenital	157 (9)	76 (48)	0.88 (0.61 to 1.26)	0.47
Circulatory	162 (10)	74 (46)	0.77 (0.53 to 1.10)	0.15
Oncology	141 (8)	52 (37)	0.53 (0.36 to 0.78)	<0.01
Gastrointestinal	68 (6)	37 (54)	1.17 (0.70 to 1.99)	0.54
Metabolic	79 (5)	25 (32)	0.46 (0.27 to 0.77)	<0.01
Genitourinary	29 (2)	16 (55)	1.18 (0.55 to 2.60)	0.67
Haematology	18 (1)	<10§	0.57 (0.20 to 1.46)	0.25
Other (including perinatal)	130 (8)	77 (60)	1.39 (0.94 to 2.08)	0.1
Unknown	124 (7)	104 (84)	5.13 (3.10 to 8.96)	<0.01
Type of service received¶				
Hospice	369 (18)	292 (79)	–	–
Specialist children’s hospital	445 (22)	319 (72)	–	–
General hospital	401 (20)	183 (46)	–	–
Community/school**	825 (30)	352 (43)	–	–

	Total population (mean)	Those seen in census week (mean)	OR (95% CI)	P value
Index of Multiple Deprivation rank††	10 005.58	10 176.08	1.0 (0.99 to 1.00)	0.99

* There were 53 (3%) children and babies with a missing value for whether they were seen or called in the census week.

† The numbers assessed using the children’s framework do not equal the total number of children in the study, as some babies (n=30) were assessed using the children’s framework and some children (n=36) were assessed using the babies’ framework. The colour-coded level of need is not reported for babies due to issues with data quality. One child did not have a level of need recorded.

‡ One child had no age recorded.

§ Percentage not provided due to censoring.

¶ The total number is higher than the number of children in the dataset, as some children received and were seen by multiple services.

** 38 patients were recorded by a school.

†† Index of Multiple Deprivation ranks for England range from 1 (the most deprived area) to 32,844 (the least deprived area).

67% of the study population were of White ethnicity, and 16% were South Asian. The 2021 North West Census profile indicates that the population of the North West is 86% White and 8% Asian.^14^ South Asian children are therefore over-represented in these data, more than would be expected from the population demographics. This is, however, in line with the higher prevalence in these minoritised ethnic groups reported in other studies.^2^

More children in the total dataset were known to the community teams (30%) than to other teams, with otherwise roughly equal proportions being reported by the hospices, specialist hospitals or general hospital services.

### Point prevalence of palliative care need

**Figure 1:**
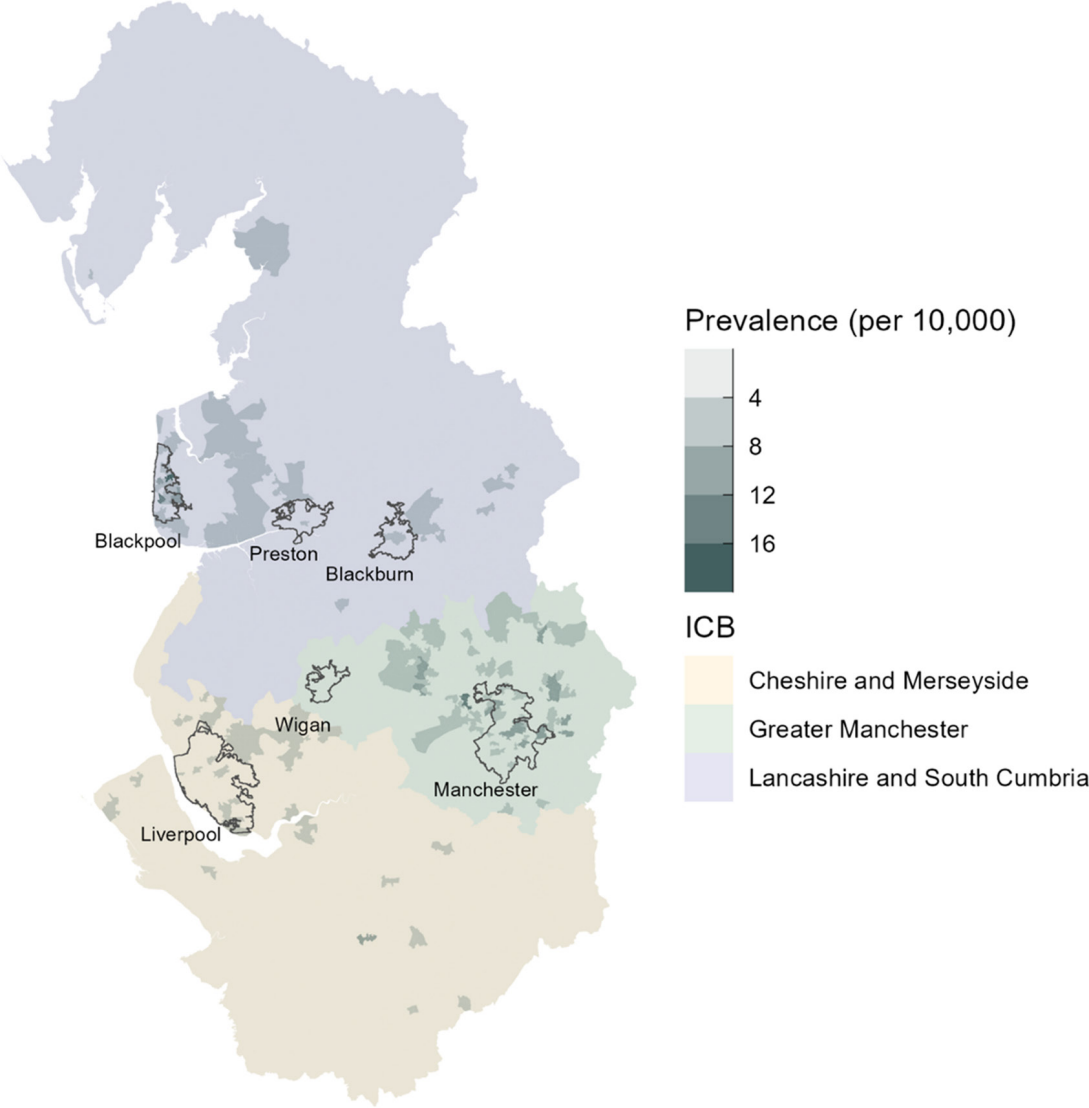
Map of prevalence of palliative care need across the North West. Darker shaded areas have a higher prevalence per 10 000 of the child population in the region. The background colour shows the Integrated Care Board (ICB) area. Exemplar city outlines are provided to aid understanding.

In the study data, 1525 patients were recorded living in the North West, and the eligible paediatric population of the region was estimated to be 1 512 514. The overall prevalence of babies’ and children’s palliative care need (a baby or child known to a service) in the North West was found to be 10.1 per 10 000 0–19-year-olds (95% CI 9.6 to 10.6). This ranged from 13.5 per 10 000 for those aged under 1 year to 7.46 per 10 000 for those aged 16–19 years (table 3). Prevalence also varied according to ethnicity, from 5.04 per 10 000 for those of mixed ethnicity to 14.5 for those of South Asian ethnicity (table 3). A map of the prevalence of palliative care need can be found in figure 1, showing that areas with higher prevalences are clustered around larger cities.

**Table 3:** Ethnicity and age group case numbers, populations and point prevalence estimates*

	Cases	Population estimates	Prevalence per 10 000
Ethnicity†			
Black	49	37 513	13.1 (9.41 to 16.72)
East Asian	42	29 281	14.3 (10.00 to 18.70)
Mixed	36	71 383	5.04 (3.40 to 6.69)
Other	49	13 194	37.1 (26.80 to 47.50)
South Asian	239	164 721	14.5 (12.70 to 16.30)
White	1059	1 224 134	8.65 (8.13 to 9.17)
Age group			
<1	117	86 853	13.5 (11.10 to 15.90)
1–5 years	449	429 493	10.4 (9.49 to 11.40)
6–10 years	462	426 464	10.8 (9.85 to 11.8)
11–15 years	402	431 547	9.32 (8.41 to 10.2)
16–19 years	244	327 176	7.46 (6.52 to 8.39)

* Numbers of cases differ from table 2 as table 3 only includes children, young people and babies who live within the three ICB areas in North West England.

† Children with ‘Unknown’ ethnicity do not appear in the table as it is not possible to estimate point prevalence for that population.

ICB, Integrated Care Board.

### Associations between patient characteristics and odds of being seen by services in the census week

Children and young people being recorded in the prognosis categories amber (months) (OR 3.70, 95% CI 2.28 to 6.15) and amber (years) (OR 2.35, 95% CI 1.73 to 3.21) were significantly more likely to be seen than those in the green category (table 4). Children aged <1 year were significantly more likely to be seen than those aged 1–5 years (OR 2.85, 95% CI 1.43 to 5.89) (table 4). Those of South Asian ethnicity were nearly half as likely to be seen during the census week compared with those from White ethnicity (OR 0.45, 95% CI 0.32 to 0.64) (table 4). Other explanatory variables investigated (deprivation, age, sex, level of need (Spectrum colour) and diagnosis category) did not contribute to this difference according to ethnicity in our modelling. While children of Black ethnicity were associated with increased odds of being seen in the census week (OR 2.16, 95% CI 1.06 to 4.64), this should be interpreted cautiously due to the small numbers in this category (table 4). Adjusted ORs for all explanatory variables are reported in online supplemental table S3.

**Table 4:** Adjusted ORs for Spectrum colour, age group and ethnicity estimated in multiple regression analysis

Category	Adjusted OR (95% CI)	P value
Spectrum colour for Children and Young People (CYP)		
Baseline: CYP green		
CYP red	1.61 (0.61 to 4.32)	0.33
CYP amber (years)	2.35 (1.73 to 3.21)	<0.01
CYP amber (months)	3.70 (2.28 to 6.15)	<0.01
CYP yellow	0.93 (0.70 to 1.24)	0.64
Age group		
Baseline: 1–5 years		
0–12 months	2.85 (1.43 to 5.89)	<0.01
6–10 years	0.66 (0.48 to 0.89)	<0.01
11–15 years	0.64 (0.46 to 0.88)	<0.01
16–18 years	0.52 (0.36 to 0.75)	<0.01
19+ years	1.25 (0.42 to 4.23)	0.70
Ethnicity		
Baseline: white		
Black	2.16 (1.06 to 4.64)	0.04
East Asian	0.96 (0.49 to 1.89)	0.91
Mixed	1.05 (0.49 to 2.23)	0.91
Other	1.01 (0.52 to 2.00)	0.97
South Asian	0.45 (0.32 to 0.64)	<0.01
Unknown	0.87 (0.53 to 1.43)	0.59

The adjusted OR compares the odds of being seen in the census week compared with the baseline category level in each category.

## Discussion

### Summary

This novel study used the Spectrum of Children’s Palliative Care Needs to estimate population-level palliative care needs. We calculated an overall point prevalence of 10.1 babies, children and young people with palliative care needs per 10 000 population. We found that children and young people recorded in the prognosis categories amber (months) and amber (years) were significantly more likely to be seen than those in the green category. Those of South Asian ethnicity were only nearly half as likely to be seen during the census week compared with those of White ethnicity. Other explanatory variables investigated, including deprivation and diagnosis category, did not contribute to this difference in our modelling.

### Strengths and limitations

The study data are comprehensive, having been contemporaneously collected by clinicians across nearly every service providing some form of generalist or specialist paediatric palliative care in North West England. They capture details of not only children who were active contacts within the census period but also a wider range of children currently on service caseloads. The training data collectors received gives a high degree of confidence that there is comparability between services in the approach to data capture. North West England is a large region incorporating metropolitan cities as well as rural and coastal areas, including areas of high and low ethnic diversity and varying levels of deprivation, enhancing the generalisability of findings beyond the study setting.

There are limitations to these data. First, the ethnicity data were captured by clinical staff without self-categorisation by parents or children. This was done against broad categories informed by staff working alongside the families. However, there is a risk that some data may have been incorrectly captured or may not match the way that families self-identify. Second, there may be inconsistencies in the way that services captured information on children not seen within the census period. This may have depended on how they defined and captured caseloads and whether they chose to collect these data. Services with particularly large caseloads may have had less capacity to capture these data. It is likely, therefore, that the total number of children with life-limiting illnesses in our geography is undercounted. There may be service patterns driving this that we are not aware of (eg, those of South Asian ethnicity are less likely to be on the caseloads of those who reported the most data). When mapping areas of high prevalence, our analysis could not account for families moving from rural to urban areas to be closer to services. While this is likely a small minority, it may have biased our interpretation of clustering.

### Comparison with existing evidence

The prevalence identified is lower than previously published national estimates; however, the published estimates were calculated per year using hospital-based diagnostic data, so they are not directly comparable.^2^ The question on expectation of survival, which those using the Spectrum tool (measure of need in our study) are asked to answer, is arguably more appropriate for identifying palliative care needs than diagnosis alone. This is because it relies more on practitioner judgement than on assuming that every child with the same diagnosis has the same need for palliative care. While there is a chance that those completing the tool did not identify every child with palliative and end-of-life care needs on their caseload, our study demonstrates that, with training, practitioners can use the Spectrum tool to estimate population-level need.

This study is part of a wider body of literature attempting to incorporate more sensitive measures of need or complexity into estimates of children’s population-level palliative care needs.^15^,^17^ Others have, for example, measured medical complexity to try to identify children with life-limiting conditions who may need extra time, expertise and resources from healthcare professionals, using diagnosis, severity, resource use, service utilisation and other patient data to estimate this.^17^ In our study, service activity was an outcome rather than a measure of need, and, having controlled for diagnosis and severity of need, we interpreted differences in service activity as inequities. However, the association of some patient characteristics with greater service use may be to do with those patients experiencing greater complexity of needs in ways that we did not measure. Future studies using the Spectrum framework or similar tools to estimate the need for paediatric palliative care services and inequities in access may want to consider including an objective measure of complexity to compare with practitioner-assessed need.

A key study finding was regarding the ethnicity of the child, with children of South Asian ethnicity being half as likely to receive a call or visit in the census week. This is aligned with existing data that identifies that those from ethnic minority populations are less likely to receive hospice care.^15 16^ Understanding why this may be is likely to be complex with structural, personal and cultural barriers.^17^,^20^ While such a disadvantage is not unique to palliative care,^21^,^24^ there is an urgency in paediatric palliative care to receive appropriate care due to the time-limited nature of care, but also the cultural and religious significance of the period surrounding death.^25^ Interactions with health and social care services have the potential to impact both bereavement experiences and later health, as well as colour ongoing attitudes towards healthcare.^26^,^28^ Such negative experiences appear to be magnified for parents from minority ethnic populations, where there is a cumulative experience of disadvantage.^29^

### Implications for practice, policy and research

This study draws attention to the need for careful and inclusive development of interventions that ensures all children and their parents, regardless of background, have access to safe, appropriate palliative care. While levels of missing data were relatively low in our study, conflicting data on ethnicity and other characteristics were sometimes input by data collectors. Research into how ethnicity and other demographic data can be collected and documented accurately, sensitively and appropriately within palliative care services would support future efforts to interrogate and respond to inequities in care. Future studies directly comparing the Spectrum tool with diagnostic-based or complexity-based methods for collecting population-level data on palliative care need could also help test the validity of this tool.

## Supplementary material

10.1136/bmjpo-2025-004069 online supplemental file 1

## Data Availability

Data may be obtained from a third party and are not publicly available.

## References

[1] Fraser LK, Miller M, Hain R (2012). Rising national prevalence of life-limiting conditions in children in England. Pediatrics.

[2] Fraser LK, Gibson-Smith D, Jarvis S (2021). Estimating the current and future prevalence of life-limiting conditions in children in England. Palliat Med.

[3] Bowers AP, Chan RJ, Herbert A (2020). Estimating the prevalence of life-limiting conditions in Queensland for children and young people aged 0-21 years using health administration data. Aust Health Rev.

[4] Lin S-C, Huang M-C (2024). Prevalence, trends, and specialized palliative care utilization in Taiwanese children and young adults with life-limiting conditions between 2008 and 2017: a nationwide population-based study. *Arch Public Health*.

[5] Serrano-Pejenaute I, Astigarraga I, López-Bayón J (2025). Pediatric complex chronic and life-limiting conditions in the Basque public health system: cross-sectional prevalence study. World J Pediatr.

[6] Burgio NM, Jennessen S (2025). Prevalence and Mortality of Life-Threatening and Life-Shortening Diseases in Children and Adolescents in Germany. Clin Pediatr (Phila).

[7] Connor SR, Downing J, Marston J (2017). Estimating the Global Need for Palliative Care for Children: A Cross-sectional Analysis. J Pain Symptom Manage.

[8] Kitreerawutiwong N, Kitreerawutiwong K, Keeratisiroj O (2024). Methods used to identify the prevalence of palliative care needs: An integrative review. *Pall Supp Care*.

[9] Delamere T, Balfe J, Fraser LK (2024). Defining and quantifying population-level need for children’s palliative care: findings from a rapid scoping review. BMC Palliat Care.

[10] Amarri S, Ottaviani A, Campagna A (2021). Children with medical complexity and paediatric palliative care: a retrospective cross-sectional survey of prevalence and needs. Ital J Pediatr.

[11] Shaw KL, Brook L, Mpundu-Kaambwa C (2015). The Spectrum of Children’s Palliative Care Needs: a classification framework for children with life-limiting or life-threatening conditions. BMJ Support Palliat Care.

[12] Office for National Statistics Lower layer super output area population estimates. https://www.ons.gov.uk/peoplepopulationandcommunity/populationandmigration/populationestimates/datasets/lowersuperoutputareamidyearpopulationestimates.

[13] Wohland PR, Norman P, Lomax N (2018). NEWETHPOP - ethnic population projections for UK local areas 2011-2061.

[14] Nomis North West region. 2021 census profile. www.nomisweb.co.uk/sources/census_2021/report?compare=E12000002.

[15] Tobin J, Rogers A, Winterburn I (2022). Hospice care access inequalities: a systematic review and narrative synthesis. BMJ Support Palliat Care.

[16] Fraser LK, Fleming T, Miller M (2010). Palliative care discharge from paediatric intensive care units in Great Britain. Palliat Med.

[17] Evans N, Meñaca A, Andrew EVW (2012). Systematic review of the primary research on minority ethnic groups and end-of-life care from the United Kingdom. J Pain Symptom Manage.

[18] Brown E, Patel R, Kaur J (2013). The South Asian culture and palliative care for children, young people, and families--a discussion paper. Issues Compr Pediatr Nurs.

[19] Piracha NZ, Nickel LB, Quryshi A (2024). Muslims and End-of-Life Healthcare in Non-Muslim Majority Nations: A Systematic Literature Review. J Pain Symptom Manage.

[20] Kent W (2020). Who supports the families of black and minority ethnic children with life-limiting conditions?.

[21] Manuel JI (2018). Racial/Ethnic and Gender Disparities in Health Care Use and Access. Health Serv Res.

[22] Fiscella K, Sanders MR (2016). Racial and Ethnic Disparities in the Quality of Health Care. Annu Rev Public Health.

[23] Yaya S, Yeboah H, Charles CH (2020). Ethnic and racial disparities in COVID-19-related deaths: counting the trees, hiding the forest. BMJ Glob Health.

[24] Javed Z, Haisum Maqsood M, Yahya T (2022). Race, Racism, and Cardiovascular Health: Applying a Social Determinants of Health Framework to Racial/Ethnic Disparities in Cardiovascular Disease. Circ Cardiovasc Qual Outcomes.

[25] Puchalski CM, O’Donnell E (2005). Religious and spiritual beliefs in end of life care: how major religions view death and dying. Tech Reg Anesth Pain Manag.

[26] Stroebe M, Schut H, Stroebe W (2007). Health outcomes of bereavement. Lancet.

[27] Dias N, Friebert S, Donelan J (2021). Bereaved Parents’ Health Outcomes Following the Death of Their Child. Clin Pract Pediatr Psychol.

[28] McNeil MJ, Baker JN, Snyder I (2021). Grief and Bereavement in Fathers After the Death of a Child: A Systematic Review. Pediatrics.

[29] Umberson D, Donnelly R (2022). The Death of a Child and Parents’ Psychological Distress in Mid to Later Life: Racial/Ethnic Differences in Exposure and Vulnerability. J Gerontol B Psychol Sci Soc Sci.

[30] Ministry of Housing, Communities & Local Government (2019). English indices of deprivation 2019: index of multiple deprivation.

