# Supplementary material for: How many children have palliative care needs and which of them are less likely to be actively seen by services? A cross-sectional analysis of survey data
Source: BMJ Paediatr Open. 2026 Apr 10;10(1):e004069. doi: 10.1136/bmjpo-2025-004069 (PMC13084903; doi:10.1136/bmjpo-2025-004069)
Supplement: online supplemental file 1 [file bmjpo-10-1-s001.pdf]

Supplementary Materials

Table S1. Criteria for deciding data values where there was conflicting data for individual children or babies. Data were imputed based on criteria in ascending order.

Criteria	Description	Variables where this criterion applied
1	If one of the duplicate values is NA or 'unknown' select other available value	Ethnicity, Sex, Diagnosis
2	For those with more than two entries, where there is a most common value, select that value	All
3	Select highest value	Age Group, Spectrum colour (i.e. highest level of need)
4	For all remaining rows that have a conflicting value, value = 'Unknown'[1]	Ethnicity, Sex, Diagnosis
[1] Numbers and percentages for number of Unknown values derived from conflicting values is presented for Ethnicity, Sex, and Diagnosis in Table S2		

Table S2. Number of children with conflicting values for Sex, Ethnicity, and Diagnosis, presented as a category level

Category	Level	N (% in category)
Sex[1]	Female	747 (44)
	Male	910 (54)
	Other	1 (0)
	Unknown – not available	3
	Unknown – conflicting data	33 (0)
Ethnicity	Black	54 (3)
	East Asian	42 (42)
	Mixed	36 (2)
	Other	53 (3)
	South Asian	268 (16)
	White	1143 (67)
	Unknown – not available	74 (4)
	Unknown – conflicting data	24 (1)
Diagnosis	Circulatory	162 (10)
	Congenital	157 (9)
	Gastrointestinal	67 (6)
	Genitourinary	29 (2)
	Haemotology	18 (1)
	Metabolic	79 (5)

	Neurology	562 (33)
	Oncology	141 (8)
	Other (inc. perinatal)	130 (8)
	Respiratory	224 (13)
	Unknown – not available	6 (0)
	Unknown – conflicting values	118 (7)
[1] Sex has been rounded to the nearest 5 to prevent disclosure of identifiable data		

Table S3. Adjusted odds ratios of multivariate analysis for all included explanatory variables. The adjusted odds ratio compares the odds of being seen in the census week compared to the baseline category level in each category

Category	Adjusted Odds Ratio (CI)	p-value
**Spectrum Colour**		
Baseline: CYP Green		
CYP Amber (Years)	2.35 (1.73, 3.21)	<0.01
CYP Amber (Months)	3.70 (2.28, 6.15)	<0.01
CYP Yellow	0.93 (0.70, 1.24)	0.64
CYP Red	1.61 (0.61, 4.32)	0.33
**Category of diagnosis**		
Baseline: Neurology		
Circulatory	0.54 (0.35, 0.83)	<0.01
Congenital	0.80 (0.54, 1.19)	0.27
Gastrointestinal	1.14 (0.64, 2.03)	0.65
Genitourinary	1.08 (0.47, 2.52)	0.85
Haematology	0.50 (0.17, 1.42)	0.20
Metabolic	0.35 (0.19, 0.63)	<0.01
Oncology	0.58 (0.38, 0.87)	0.01
Other	1.24 (0.79, 1.97)	0.35
Respiratory	0.66 (0.45, 0.95)	0.03
Unknown	4.80 (2.60, 9.49)	<0.01
**Ethnicity**		
Baseline: White		
Black	2.16 (1.06, 4.64)	0.04
East Asian	0.96 (0.49, 1.89)	0.91
Mixed	1.05 (0.49, 2.23)	0.91
Other	1.01 (0.52, 2.00)	0.97
South Asian	0.45 (0.32, 0.64)	<0.01

Unknown	0.87 (0.53, 1.43)	0.59
**Index of Multiple Deprivation Rank**	1.00 (1.00, 1.00)	0.83
**Age group**		
Baseline: 1-5 years		
0-12 months	2.85 (1.43, 5.89)	<0.01
6-10 years	0.66 (0.48, 0.89)	<0.01
11-15 years	0.64 (0.46, 0.88)	<0.01
16-18 years	0.52 (0.36, 0.75)	<0.01
19+ years	1.25 (0.42, 4.23)	0.70
**Sex**		
Baseline: Female		
Male	1.00 (0.80, 1.25)	0.98
Other/Unknown	0.72 (0.28, 1.97)	0.51
