# Supplementary material for: How many children have palliative care needs and which of them are less likely to be actively seen by services? A cross-sectional analysis of survey data
Source: BMJ Paediatr Open. 2026 Apr 10;10(1):e004069. doi: 10.1136/bmjpo-2025-004069 (PMC13084903; doi:10.1136/bmjpo-2025-004069)
Supplement: Supplementary file 2 [file bmjpo-2025-004069.reviewer_comments.pdf]

PEER REVIEW HISTORY

BMJ Paediatrics Open publishes all reviews undertaken for accepted manuscripts. Reviewers are asked to complete a checklist review form and are provided with free text boxes to elaborate on their assessment. These free text comments are reproduced below.

ARTICLE DETAILS

**TITLE (PROVISIONAL)**	How many children have palliative care needs and which of them are less likely to be actively seen by services? A cross-sectional analysis of survey data
**AUTHORS**	French, Maddy<br>Walshe, Catherine<br>Garner, Alex

VERSION 1 – REVIEW

**REVIEWER NAME**	Lin, Shih-Chun
**REVIEWER AFFILIATION**	Kaohsiung Medical University, School of Nursing
**REVIEWER CONFLICT OF INTEREST**	No Conflict of Interest
**AI DISCLOSURE**	No, I have not used AI
**DATE REVIEW RETURNED**	13-Oct-2025

**GENERAL COMMENTS**	Thank you for the opportunity to review this manuscript.<br>1. On Page 5, while the authors included all services known to the Northwest Regional Children and Young People's Palliative Care Network, how did they determine which services were related to palliative care versus general services for children with chronic illnesses or disabilities, such as special school provision?<br>2. The methods section should be enhanced by including a definition of the spectrum of children's palliative care needs and specifying who assessed the levels of need for the children involved in the study.<br>3. On Page 7, Table 1 contains a small "5" next to the word "colour," which is unclear in meaning. Additionally, it mentions "see Appendix 1 for full descriptors," but there is no Appendix 1 attached.<br>4. On Page 11, Table 2 shows inconsistent totals for each characteristic. The sum of the percentages for the types of services received does not equal 100%. This needs to be rechecked.<br>5. On Page 12, participants were categorized into one diagnostic category. Were any participants classified under two or more diagnostic categories? If so, how did the authors decide which category to assign?<br>6. On Page 13, there are inconsistencies in the total number of cases between ethnicity and age groups.<br>7. On Page 13, mark number 5 should be placed above the word in the table.<br>8. In Table S3, if multivariate analysis was used, the methods section should specifically state which variables were controlled. Additionally, using "adjusted odds ratio" instead of "odds ratio" in both Table S3 and the text will enhance clarity.<br>9. Finally, Pages 13 and Figure 1 indicate that areas with higher prevalence are clustered around larger cities. Is it possible that most children in rural areas are transported to larger cities for services? This could be a limitation of the study.

VERSION 1 – AUTHOR RESPONSE

Thank you for the opportunity to review this manuscript.<br><br>1. On Page 5, while the authors included all services known to the Northwest Regional Children and Young People's Palliative Care Network, how did they determine which services were related to palliative care versus general services for children with chronic illnesses or disabilities, such as special school provision?	Thank you for this comment. The determination we were seeking was whether a child had palliative care needs rather than whether the service itself was a 'palliative care' service. Many services that provided data were not 'palliative care' services. For example, wards in hospitals from which we collected data were not 'palliative care' wards. The individual who completed the data extraction form for each site indicated in the 'data collection site' and the name of the relevant team or ward. We used this information, in consultation with the Network collaborators to determine type of service that had identified the child with palliative care needs.
2. The methods section should be enhanced by including a definition of the spectrum of children's palliative care needs and specifying who assessed the levels of need for the children involved in the study.	Thank you for this suggestion. We agree and have made the following amendments to the text. The highlighted text is text that has been moved from the Background to the Methods section:<br><br>**Added to Page 4-5:**<br><br>The Spectrum of Children's Palliative Care Needs is a classification framework for children with life-limiting or life-threatening conditions. It is designed for multi-disciplinary teams to assess the likely prognosis of a child and group them accordingly, ranging from Green where survival to adulthood is considered likely to Red where survival is not expected beyond the next few weeks.<br><br>**Added to Page 6:** A clinician within the Network was nominated to lead data collection in each site. Where clinicians knew the child for whom they were inputting data and were confident estimating their prognosis they would complete this field themselves, otherwise consult with another clinician in that team who could provide that information.
3. On Page 7, Table 1 contains a small "5" next to the word "colour," which is unclear in meaning. Additionally, it mentions "see Appendix 1 for full descriptors," but there is no Appendix 1 attached.	Thank you for spotting this. This was an error and has been removed.
4. On Page 11, Table 2 shows inconsistent totals for each characteristic. The sum of the percentages for the types of services received does not equal 100%. This needs to be	Thank for you highlighting the inconsistencies and need for clarity here. We re-checked this table and identified that for the Spectrum colour code, Age, and Sex, the totals did not equal the total dataset population. The reasons for this, and any amendments made, are as follows:<br><br>*Spectrum colour code:* We only report the Spectrum colour codes that were provided by data collectors using the children's version of the

rechecked.	Spectrum framework. This version was sometimes used to assess babies (<1 years) and conversely the babies framework was sometimes used to assess a child's (>1 years) needs. This means that the totals for this category do not add up to the total number of children (>1 years) in the study. We have added a footnote to Table 2 explaining this and added the totals for babies and children in the dataset in the narrative to demonstrate that they sum to the total study population.<br><br>**Correction and footnote added to Table 2**<br><br>**Totals for babies and children added to Page 9**<br><br>*Age*: One child did not have age recorded which explains the discrepancy in the total figure for this category. We have clarified this in a footnote.<br><br>**Footnote added to Table 2.**<br><br>*Sex:* We have added the number and % for the Other/Unknown category to make it clear that the total number in the category equals the number in the total dataset. We have also corrected an error in the breakdown of the 'Unknown' category provided in the Supplementary Table 2<br><br>**Correction to Table 2**<br><br>**Correction to Supplementary Table 2**
5. On Page 12, participants were categorized into one diagnostic category. Were any participants classified under two or more diagnostic categories? If so, how did the authors decide which category to assign?	Thank you for this comment. Criteria for deciding data values where there was conflicting data, including instances of multiple diagnoses, is provided in Supplementary Table 1. The number of children with conflicting values for diagnosis is provided in Supplementary Table 2.
6. On Page 13, there are inconsistencies in the total number of cases between ethnicity and age groups.	Thank you for highlighting this. This is because children with 'Unknown' ethnicity do not appear in that table as it is not possible to estimate population point prevalence of that population. We have added a sentence to the footnote of Table 3 on Page 13 clarifying that point.<br><br>**Footnote in Table 3 amended**
7. On Page 13, mark number 5 should be placed above the word in the table.	Thank you for this. However, we could not see a mark number 5 on Page 13. We did see that the number 5 in the footnote of the Table 2 on Page 12 was not in subscript when it should have been. We have now amended this. We hope this has addressed this comment but if not, please let us know.<br><br>**Amendment made to footnote in Table 2**

8. In Table S3, if multivariate analysis was used, the methods section should specifically state which variables were controlled. Additionally, using "adjusted odds ratio" instead of "odds ratio" in both Table S3 and the text will enhance clarity.	Thank you for this comment. We have amended 'odds ratio' to 'adjusted odds ratio' as appropriate in the text.<br><br>**Amendments made to Table S3**<br><br>**Amendments made to Page 8 and Page 14**<br><br>The following section in Methods addresses the first point in this comment regarding variables in the multiple logistic regression. Note that for our adjusted analysis we used a multiple logistic regression not a multivariate analysis (https://academic.oup.com/ntr/article-abstract/23/8/1446/5812038):<br><br>Univariate models were first fitted, then a multiple logistic regression model was fitted containing all variables included in each univariate model. Explanatory variables that were tested were spectrum of palliative care needs colour, diagnosis category, ethnicity, index of multiple deprivation, age, and sex.
9. Finally, Pages 13 and Figure 1 indicate that areas with higher prevalence are clustered around larger cities. Is it possible that most children in rural areas are transported to larger cities for services? This could be a limitation of the study.	Thank you for this suggestion which is an important consideration.<br><br>As a point of clarity, the map was generated using the residence of the child rather than the location of a service. We used the postcode for the child's main residence to estimate rurality. While there may be some children whose families move to be closer to services in larger cities we anticipate that this is a small minority and is unlikely to bias estimates. However, we have included this as a consideration in our limitations as it is something we are unable to control for specifically in the analysis.<br><br>**Added to pages 15-16:** When mapping areas of high prevalence, our analysis could not account for families moving from rural to urban areas to be closer to services. While this is likely a small minority, it may have biased our interpretation about clustering.

VERSION 2 – REVIEW

**REVIEWER NAME**	Lin, Shih-Chun
**REVIEWER AFFILIATION**	Kaohsiung Medical University, School of Nursing
**REVIEWER CONFLICT OF INTEREST**	No Conflict of Interest
**AI DISCLOSURE**	No, I have not used AI
**DATE REVIEW RETURNED**	01-Feb-2026

**GENERAL COMMENTS**	Thank the authors for revising the manuscript and for their detailed explanation.
